# Acetylation of Tau Induces Alzheimer's Disease-Associated Tau in Transgenic Mice

**DOI:** 10.1093/geroni/igab046.3457

**Published:** 2021-12-17

**Authors:** Addison Ali, Kristeen Pareja, Tara Tracy

**Affiliations:** 1 University of California, Davis, Martinez, California, United States; 2 Buck Institute, Buck Institute, California, United States; 3 Buck Institute for Research on Aging, Novato, California, United States

## Abstract

Alzheimer’s disease (AD) is a neurodegenerative disorder that is characterized by neurofibrillary tangles (NFTs) and amyloid beta plaques. These NFTs are made up of aggregated tau proteins. Tau is involved in stabilizing microtubules and does not usually display aggregation. Acetylation of tau protein causes an increase in tau aggregation but its role in AD progression is still not well understood. I hypothesized that enhanced acetylated tau results in an increase in AD-like tau pathology. To test this, a murine prion promoter-tauKQ transgene was injected into the mouse fertilized oocyte. The tauKQ mutation alters lysine to glutamine to mimic acetylation of tau. Nontransgenic mice were used as controls. AT8 and GT-38 antibodies were used in immunohistochemistry (IHC) to target phosphorylated tau and AD-associated tau, respectively. GT-38 is conformation-dependent and requires 3R and 4R tau isoforms which makes it specific to AD. Through immunofluorescence, increased phosphorylated tau was observed in the hippocampus of the tauKQ mice compared to the nontransgenic mice. I focused on the dentate gyrus, CA1 region, and the mossy fibers of the CA3 region since they are involved in many memory processes. Through chromogenic IHC, the tauKQ mice exhibited more 3R+4R tau isoform pathology in the mossy fibers than the nontransgenic mice. This data suggests that an acetylation mimic is sufficient to stimulate an abundance of AD-related tau pathology in transgenic mice which is consistent with my hypothesis. The tauKQ mouse model can assist in understanding the role of tau acetylation and tau progression for AD.

